# The relationship between depressive symptoms, health service consumption, and prognosis after acute myocardial infarction: a prospective cohort study

**DOI:** 10.1186/1472-6963-8-200

**Published:** 2008-09-30

**Authors:** Paul A Kurdyak, William H Gnam, Paula Goering, Alice Chong, David A Alter

**Affiliations:** 1 Institute for Clinical Evaluative Sciences (ICES), 2075 Bayview Avenue, Toronto, Ontario, Canada; 2 Department of Psychiatry, University of Toronto, Toronto, Ontario, Canada; 3 Health Systems Research and Consulting Unit, Centre for Addiction and Mental Health, 33 Russell Street, Toronto, Ontario, Canada; 4 Department of Health Policy, Management and Evaluation, University of Toronto, Toronto, Ontario, Canada; 5 Division of Cardiology and the Li Ka Shing Knowledge Institute, St. Michael's Hospital, Toronto, Ontario, Canada; 6 Department of Medicine, University of Toronto, Toronto, Ontario, Canada

## Abstract

**Background:**

The use of cardiovascular health services is greater among patients with depressive symptoms than among patients without. However, the extent to which such associations between depressive symptoms and health service utilization are attributable to variations in comorbidity and prognostic disease severity is unknown. This paper explores the relationship between depressive symptoms, health service cardiovascular consumption, and prognosis following acute myocardial infarction (AMI).

**Methods:**

The study design was a prospective cohort study with follow-up telephone interviews of 1,941 patients 30 days following AMI discharged from 53 hospitals across Ontario, Canada between December 1999 and February, 2003. Outcome measures were post discharge use of cardiac and non-cardiac health care services. The service utilization outcomes were adjusted for age, sex, income, comorbidity, two validated measures of prognosis (cardiac functional capacity and risk adjustment severity index), cardiac procedures (CABG or PTCA) and drugs prescribed at discharge.

**Results:**

Depressive symptoms were associated with a 24% (Adjusted RR:1.24; 95% CI:1.19–1.30, P < 0.001), 9% (Adjusted RR:1.09; 95% CI:1.02–1.16, P = 0.007) and 43% (Adjusted RR: 1.43; 95% CI:1.34–1.52, P < 0.001) increase in total, cardiac, and non-cardiac hospitalization days post-AMI respectively, after adjusting for baseline patient and hospital characteristics. Depressive-associated increases in cardiac health service consumption were significantly more pronounced among patients of lower than higher cardiac risk severity. Depressive symptoms were not associated with increased mortality after adjusting for baseline patient characteristics.

**Conclusion:**

Depressive symptoms are associated with significantly higher cardiac and non-cardiac health service consumption following AMI despite adjustments for comorbidity and prognostic severity. The disproportionately higher cardiac health service consumption among lower-risk AMI depressive patients may suggest that health seeking behaviors are mediated by psychosocial factors more so than by objective measures of cardiovascular risk or necessity.

## Background

Available evidence has demonstrated that health service consumption is higher among patients with depression than those without. In ambulatory care settings, depression is associated with a 50% increase in general medical service use, even after adjusting for age, sex and chronic medical comorbidities[1,2]. Similar depression-related service consumption patterns have been described among cohorts with cardiovascular disease[3,4].

Some authors advocate that increased health service consumption among cardiac-specific patients with depression is appropriate and concordant with their underlying cardiovascular prognosis[5]. However, others contend that depressed patients seek more health care services regardless of illness severity. Evidence from utilization patterns of depressed patients in primary care settings suggest that depressed patients use more health care services than non-depressed patients regardless of medical illness severity [6]. Few studies have quantified the relationship between depressive symptoms, illness severity, and health service consumption.

Accordingly, the objective of our study was to evaluate the impact of depressive symptoms on health service consumption and cardiovascular prognosis following AMI. AMI serves as a useful test case because the natural history of the disease has been well-described[7] and validated measures of cardiac prognostic risk severity exist[8]. Yet, unexplained variations in health service consumption exist across patient populations[9,10]; similar health service consumption variations may be explained by psychosocial factors. Because Canada's federal-provincial Medicare plan covers medically necessary services based on need rather than affordability[11], differences in health service consumption are more likely to reflect differences in health service behaviors than in a system where services are provided based on an ability to pay [3]. We hypothesized that health service consumption following AMI would be increased among patients with depressive symptoms as compared to those without and would be independent of comorbidity and cardiac illness severity.

## Methods

### Health system context

Canada's universal health insurance system provides comprehensive coverage for most medical and hospital services without user fees at point of service. Under such provisions, patients are entitled to equitable access to health care services based on medical need, regardless of age, financial status, or financial circumstances[11].

### Data source and study sample

This study is a sub-study of the Socio-Economic and Acute Myocardial Infarction Study (SESAMI) study, a prospective observational study of patients who were hospitalized for AMI throughout Ontario, Canada[10]. Patients were included if they were English-speaking and if 2 of 3 AMI criteria were met: presence of symptoms, abnormal electrocardiographic findings, or elevated serum levels of cardiac enzymes. Patients were excluded if they were younger than 19 years of age or older than 101 years of age, lacking a valid health card number issued by the province of Ontario, and those who were transferred to the recruiting hospital. Eligibility for the study required completion of a self-administered baseline survey; patients who died within 24 hours of admission, who had very severe illness, who had language barriers, or who underwent early discharge or transfer were therefore ineligible. Data came from four sources: 1) a baseline survey; 2) chart abstraction from index AMI; 3) a one-month follow-up phone survey; and 4) linked health administrative data. From 2829 patients with successfully abstracted index AMI admissions and linked health administrative data, 888 (31%) subjects did not complete the one-month follow-up survey either because of death prior to the survey (N = 73; 2%) or a refusal to participate (N = 815; 29%). A total of 1941 (69%) patients remained available for analysis. The SESAMI cohort has been described in detail elsewhere[10]. The Sunnybrook Health Sciences Centre Review Committee approved this study and all subjects gave informed consent to participate.

### Depressive symptom measures

The SESAMI survey consisted of psychometric questions that explored various domains related to depression, including low mood, loss of interest, sleep disturbance, reduced appetite/wt loss, agitation/slowing, low self-esteem/guilt, suicidal thoughts, reduced energy level, loss of concentration (Table 1). The SESAMI surveys were telephone administered by standardized trained health care personnel (nurses).

**Table 1:** BCDRS* items (included and missing) and replacement items from SESAMI survey

**Depression Questions**	**Item Questionnaire**	**Depression Construct**
I am losing weight.	BCDRS	Loss of appetite/weight loss
I have dropped many of my interests and activities.	BCDRS	Loss of interest
It must be obvious that I am disturbed and agitated.	BCDRS	Psychomotor agitation.
I am miserable or often feel like crying.	BCDRS	Low mood
I often wish I were dead.	BCDRS	Suicidal thoughts.
I feel in good spirits.	BCDRS	Low mood.
I still enjoy my meals.	BCDRS	Loss of appetite.
I get hardly anything done lately.	BCDRS	Low motivation.
I am exhausted much of the time.	BCDRS	Low energy.
My sleep is restless and disturbed.	BCDRS (missing)	Disturbed sleep
I can concentrate easily when reading the papers.	BCDRS (missing)	Poor concentration
I feel worthless and ashamed about myself.	BCDRS (missing)	Low self-esteem

**Replacement Items**		

During the past week how much of the time have you had trouble sleeping for example, having trouble falling asleep or waking up too early and unable to get back to sleep?*	GUSTO Trial Psychological Well-being Scale[14]	Disturbed sleep
During the past week, how much of the time have you had trouble concentrating or keeping your mind on what you're doing?*	GUSTO Trial Psychological Well-being Scale[14]	Poor concentration
You are a burden on others	SF-12	Low self-esteem

*The BCDRS and SF-12 items were dichotomous (yes/no). The GUSTO trial items had 4 options from None of the time, some of the time (1–2 days/wk), some of the time (3–4 days/wk), and All/most of the time (5 or more days/wk). The GUSTO items were dichotomized around the median of 3 or more days/wk.

Nine of twelve questions were directly abstracted from the Brief Carroll Depression Rating Scale (BCDRS). These nine questions served as our primary determinant of depressive symptoms. The original 12-item BCDRS is a depression rating scale which has been validated among hospitalized mentally ill populations, and has a sensitivity of 92% and a specificity of 89% using a cut-off scale of 6 [12].

While a score of 6 or more of the original 12-item BCDRS has been used to define depression, affirmative responses to 5 or more of the 9 administered BCDRS items were used to define patients as having "depressive symptoms" for the purposes of our study. A score of 5 rather than 6 was used because it corresponded most closely with the median score of 6, and because five or more depressive constructs are required to fulfill DSM-IV criteria for depression, and hence is concordant with the diagnostic criterion threshold from DSM-IV [13].

The three questions comprising the 12-item BCDRS not included in the SESAMI survey, related to sleep disturbance, concentration, and self-esteem, and were as follows:: "my sleep is restless and disturbed"; "I can concentrate easily when reading the paper"; and "I feel worthless and ashamed about myself". However, these missing questions were replaced with the following: "During the past week, how much time have you had trouble sleeping for example, having trouble falling asleep or waking up too early and unable to get back to sleep"; "During the past week, how much of the time have you had trouble concentrating or keeping your mind on what you're doing?"; and "You are a burden on others" (Table 1). These three items were abstracted from two data sources: The first two replacement items originated from a depression measure incorporated within the Global Utilization of Streptokinase and t-PA for Occluded Coronary Arteries (GUSTO) trial quality of life substudy [14], whose design served as the foundation for the SESAMI[15]. The remaining replacement item originated from the SF-12. For scoring purposes, responses related to the first 2 replacement questions were dichotomized around the median (3+ days per week vs. < 2 days per week). The last replacement question elicited a binary response (Table 1). These three additional replacement questions were analyzed separately from the 9-item BCDRS and reported within the sensitivity analysis section below.

### Demographic factors

Demographic factors such as age, sex, and income tertile were acquired from the baseline survey and were based on patient self-report.

### Cardiovascular risk severity

Two validated measures of cardiovascular risk-severity were used in this study:

The first measure was the Global Registry of Acute Coronary Events (GRACE) prognostic index. The GRACE prognostic index provides a score that reflects the probability of dying within 6 months post-AMI based on age, development (or history) of heart failure, peripheral vascular disease, systolic blood pressure, Killip class, initial serum creatinine concentration, elevated initial cardiac markers, cardiac arrest on admission, and ST segment deviation[8]. The GRACE prognostic index has been validated among SESAMI patients[16]. In addition to the GRACE prognostic index, we examined other cardiovascular risk factors, which included diabetes, hypertension, hyperlipidemia, and current or former cigarette use. Smoking history was ascertained by questionnaire. Major cardiovascular risk factors were determined by reviewing diagnostic fields in computerized hospital discharge abstracts from April 1, 1988 to the index hospitalization date using all primary and secondary ICD-9 and ICD-10 (where applicable) discharge codes. Agreement between self-reported risk factors and chart audit range was between 73% (hyperlipidemia) and 95% (diabetes) [10].

The second measure was the Duke Activity Status Index (DASI), administered to all participants. The DASI is a self-report measure validated to determine functional capacity which correlates well with peak oxygen uptake[17], one of the most important single predictors of long-term cardiovascular survival across populations[18].

### Preexisting noncardiovascular comorbid conditions

Noncardiovascular risk factors consisted of all co-morbid diseases that were captured through primary and secondary diagnostic fields of hospital discharge abstracts (Canadian Institute for Health Information) from 1 April 1988 to the presenting hospitalization. This method has been used and demonstrated to increase the prevalence of chronic conditions, which historically are known to be under-coded using single data sources alone[19,20]. Furthermore, supplementing clinical data sources with longer retrospective ascertainment of comorbidities using administrative data has been shown to improve accuracy [21]. Non-cardiovascular conditions were categorized as cancer and as diseases of the central nervous system, endocrine system, hematology system, musculoskeletal system, respiratory system, gastrointestinal system, and genitourinary system. We categorized diabetes, secondary hypertension, and hyperlipidemia as cardiovascular risk factors, not as diseases of the endocrine system[22]. Available evidence has demonstrated that the number of non-cardiac comorbidities have independent prognostic significance in patients with cardiovascular illness[23]. For the purposes of our study, non-cardiac comorbidities were analyzed as a count variable (0, 1, 2 or 3 or more). However, a re-analysis of our data in which we incorporated both the count, and the type of non-cardiac comorbidity did not alter our results.

### Early peri-infarction procedure and medication use

All of our outcomes could be influenced by whether or not a patient has received revascularization by angioplasty or coronary bypass surgery. All revascularization procedures occurring within 30 days of discharge from the index AMI hospitalization were recorded within the Ontario Health Insurance Program database. In addition, we examined the influence of cardiovascular medication (Beta-blockers, statins, ACE inhibitors, and nitrates) prescribed at discharge from index admission as acquired from chart abstraction.

### Outcomes

Our primary outcomes included cumulative outpatient physician and emergency room visits, the number and duration of recurrent hospitalizations, as well as total hospitalizations and ambulatory physician visits over the 18 months following index AMI hospitalization discharge. These outcomes were chosen because they reflect health care consumption measures that can be accurately measured in the linked administrative health data sets. Secondary outcomes included mortality, recurrent AMI, as well as time to first re-admission for cardiac-specific readmissions as general measures of prognosis. We examined recurrent AMI hospitalizations separately from other cause-specific admissions because recurrent AMI represents a deleterious prognostic outcome indicator. Moreover, unlike our other hospitalizations which are based on more discretionary criteria, recurrent AMI admissions are based on more standardized, objective clinical, laboratory, and ECG parameters. However, a re-analysis in which recurrent AMI was categorized together with the other health service consumption variables did not alter our results. Recurrent AMI was defined using the most-responsible diagnosis field of ICD-9 and ICD-10[24]. Cardiac specific re-admissions were ascertained using sets of the CIHI most-responsible diagnosis codes (AMI: ICD-9 410, 412, 4141; ICD-10 I21, I22, and I2382; angina: ICD-9 411, 413, 4140, and 4142–4149; ICD-10 I20, I241, I251, I252, I253, and I258; CHF: ICD-9 428, 415, 4254, 4298; ICD-10 I50, I255, I420, I429) that a previous study has shown have modest sensitivity, but high specificity[24].

### Statistical analysis

The Mantel-Haenszel test for trend was used for categorical data and t-tests (or nonparametric tests where relevant) were used for continuous data to detect unadjusted differences in baseline characteristics. We estimated Poisson regression models for rates of service utilization, as well as Cox proportional hazard models for mortality, recurrent AMI, and time to first angina hospitalization, adjusting for age, sex, income, cardiac risk factors, total medical comorbidities, prognostic index (GRACE score[8] and DASI[17]), drugs at discharge, and peri-infarction procedures using non-parsimonious modeling. Formal diagnostic testing revealed no evidence of multi-collinearity in any of our statistical models. We tested for violations of the proportionality assumption in all proportional hazard model specifications. Finally, for the health service utilization multivariable models, we used generalized estimating equations (GEE) to adjust for hospital-level variations in patient care.

All analyses were performed using SAS statistical software, version 9.1 (SAS Institute, Cary, NC).

### Sensitivity analyses

We conducted a number of sensitivity and sub-group analyses. First, we examined the relationship between the 3 replacement questions and health service utilization. Second, we examined the use of different cut-off scores to define "depressive symptoms" for both the 9-item BCDRS and the 3 depressive-symptom replacement items. Finally, multiple imputation was used to impute depression measures for the 888 missing depression values due to non-response[25] to test whether our outcomes were affected by systematic differences between survey responders and non-responders. Variables used to model the missing data included demographic variables (age, sex, SES), cardiac risk factors and illness severity (GRACE prognostic index and DASI), revascularization procedures, drugs prescribed at discharge, and the 9-item depression scale. There was no difference between aggregated results from 5 and 10 imputed data sets; results from 5 imputed data sets are reported. The missing depression measures were imputed using PROC MI and the results from the multiple, imputed data sets were aggregated using PROC MIAnalyze in SAS version 9.1 (SAS Institute, Cary, NC).

## Results

### Baseline characteristics

The 888 (31%) patients who did not respond to the one month follow-up survey were similar to the 1941 subjects in terms of gender, cardiac risk factors, likelihood of receiving revascularization procedures within 30 days of discharge, and the likelihood of receiving ACE inhibitors, Beta blockers, and nitrates upon discharge. However, the sample of 888 survey non-respondents were older (mean (SD) age of respondents 66.3 (13.7) years vs. 62.4 (12.8) years; P < 0.001), more likely to have 3 or more non-cardiac medical comorbidities (55.7% vs. 47.2%; P < 0.001), less likely to receive statins upon discharge (48.7% vs. 54.9%; P = 0.002), and had a higher GRACE score (predictive of 6 month mortality)(4.1 vs. 3.2; P < 0.001) than the 1941 survey respondents who consented and completed the 1 month post-myocardial infarction evaluation.

Among the 1941 subjects included in this study, the median age was 64 years (range, 26 to 96 years). 575 (29.6%) were women. Table 2 illustrates that the baseline characteristics of patients reporting five or more depressive symptoms differed from those reporting fewer depressive symptoms. Specifically, patients with depressive symptoms were more likely to be female, were less affluent, and were more likely to have diabetes, hypercholesterolemia, and non-cardiac comorbidities. There were no significant differences in baseline cardiovascular prognosis (GRACE scores 109.5 vs. 113.0, p = 0.22), but patients with depressive symptoms had poorer cardiovascular functional status (DASI score 11.2 vs. 19.6, p < 0.001; lower score indicates worse peak oxygen uptake)(Table 2).

**Table 2:** Baseline Characteristics

	Depressed*	**Non-Depressed**	**P Value**
**Characteristics**	(n = 494)	(n = 1447)	

**Age, y -- %**			0.002
19–49	21	15	
50–64	39	34	
65–74	20	29	
> 74	20	21	
**Male Sex -- %**	63	73	<0.001
**Income, Canadian $ -- %**			<0.001
Low (<$30,000)	33	24	
Intermediate ($30,000 – $59,999)	34	35	
High (>$59,999)	33	42	
**Coronary Risk Factors -- %**			
Diabetes	29	22	0.001
Hypercholesterolemia	45	39	0.02
Hypertension	49	46	0.33
Smoking	43	39	0.08
**Non-cardiac comorbidities -- %**			
None	6	9	<0.001
1	16	23	
2	27	23	
3 or more	51	46	
**Prognostic Indicators -- mean (SD)**			
GRACE 6-month prognostic index score⌷	11.6 (30.3)	113.6 (28.5)	0.22
DASI Score§	11.2 (8.4)	19.6 (11.8)	<0.001
**Processes of Care -- %**			
Percutaneous transluminal coronary angiography	8	8	0.97
Coronary angioplasty bypass grafting	12	10	0.28
ACE Inhibitor	63	62	0.64
Beta Blocker	68	70	0.44
Statin	55	55	0.89
Nitrate	37	31	0.01

*The depression measure is a depression scale containing 9 items from the Brief Carroll Depression Scale (BCDS)(cut-off score of 5).

⌷Abbreviation: GRACE: Global Registry of Acute Coronary Events scale.

§Abbreviation: DASI: Duke Activity Status Index

### Health service consumption

Total number of hospitalizations, total number of hospitalization days, length of hospital stays, and post-AMI ambulatory service use were greater for patients with depressive symptoms (Table 3).

**Table 3:** Health service consumption in depressed and non-depressed post-AMI patients.

**Service Consumption Variable**	Depressed*	**Non-depressed**	**P Value**
**Hospitalization -- mean (SD)**			
Total hospitalization days⌷	8.5 (19.8)	5.5 (14.6)	0.002
Total cardiac hospitalization days⌷	4.8 (10.2)	3.3 (7.6)	0.002
Total non-cardiac hospitalization days⌷	3.7 (15.5)	2.3 (11.5)	0.06
Total number of hospitalizations	1.2 (1.7)	0.8 (1.2)	<0.001
Total number of cardiac hospitalizations	0.7 (1.1)	0.5 (0.9)	<0.001
Total number of non-cardiac hospitalizations	0.5 (1.1)	0.3 (0.7)	0.001
**Ambulatory service consumption -- mean (SD)**			
Cardiologist visits	12.5 (11.5)	9.8 (10.2)	<0.001
General internist visits	14.1 (26.0)	10.8 (20.9)	0.01
Family doctor visits	36.5 (25.3)	31.6 (24.6)	<0.001
Emergency department visits	1.7 (2.3)	1.3 (1.9)	<0.001

*The depression measure is a depression scale containing 9 items from the Brief Carroll Depression Scale (BCDS)(cut-off score of 5).

⌷Hospitalization days are a count of total days in hospital over the 18-month follow-up period and can accumulate from multiple hospitalizations.

**Figure 1:**
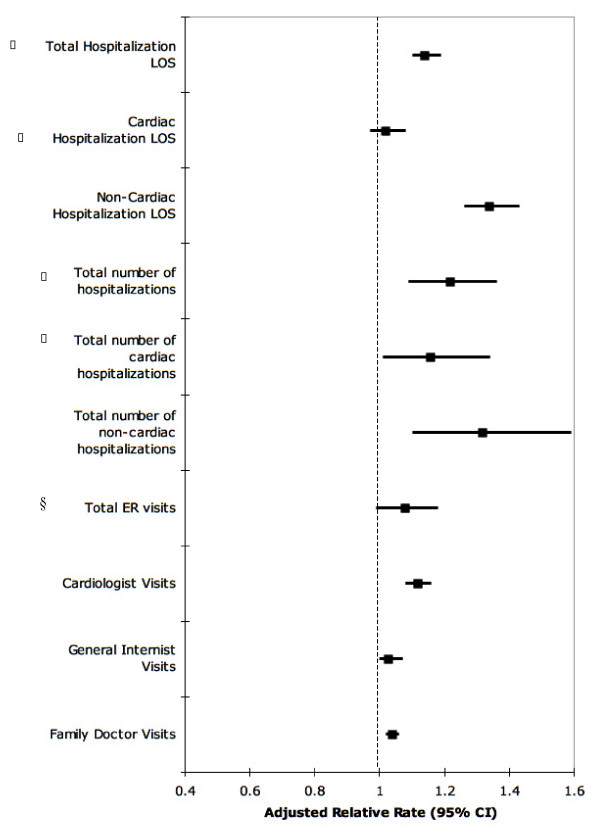
The adjusted relative rate of health service consumption attributable to depression.* All outcomes were adjusted for age, sex, income, cardiac risk factors, coronary artery bypass graft (CABG), percutaneous, transluminal coronary angiography (PTCA), drugs at discharge, GRACE prognostic index score, and DASI score. Hospitalization days are a count of total days in hospital over the 18-month follow-up period and can accumulate from multiple hospitalizations. * The depression measure is a depression scale containing 9 items from the Brief Carroll Depression Scale (BCDS)(cut-off score of 5). ⌷ Total and cardiac hospitalization results excluded recurrent AMI hospitalizations. § Abbreviation: ER – Emergency Room.

After adjustment for age, sex, income, risk factors, medical comorbidity, prognosis (GRACE score), drugs at discharge, 30-day procedure use (percutaneous coronary interventions and/or coronary artery bypass surgery), and symptom burden (DASI), depressive symptoms remained an independent predictor of most service consumption measures, with a 24% (Adjusted RR:1.24, 95% CI:1.19–1.30, P < 0.001) increase in all cause re-hospitalizations, a 9% (Adjusted RR:1.09; 95% CI:1.02–1.16, P = 0.007) increase in cardiac-related hospitalizations and a 43% (Adjusted RR:1.43; 95% CI:1.34–1.52, P < 0.001) increase in non-cardiac hospitalizations visits following AMI discharge (Figure 1). Depressive symptoms were associated with increases in cardiology, internal medicine and family practice visits after adjustment for baseline variables (Figure 1).

Cardiac-specific re-admissions were comprised mostly of angina and CHF hospitalizations. Depressed patients were substantially more likely to be admitted for angina in the 18 months post-AMI than non-depressed patients (HR 1.75; 95% C.I. 1.44 – 2.14), even when the DASI is included in the model (HR 1.41; 95% C.I. 1.14–1.75). However, after adjusting for emergency-room visits, depression was no longer a significant predictor of hospital readmissions.

To evaluate whether depressive-associated increases in health service consumption were consistent across cardiac illness risk severity levels, subgroup analyses were performed in which the sample was stratified according to median GRACE prognostic index score and median DASI scores. When stratifying patients around the median GRACE or DASI scores, the increase in health service consumption associated with depressive symptom burden was disproportionately higher among patients with lower GRACE scores (better cardiac prognostic risk) and higher DASI scores (greater functional capacity) than among their higher risk poorer functional capacity counterparts (Table 4).

**Table 4:** The relative risks of health service utilization among those with (vs. without) depression after adjustment for baseline factors stratified by prognostic risk and functional capacity (among the entire AMI cohort, and among those in which death and recurrent AMI were excluded).*

	Among the entire sample		Among those without death or re-infarction	
	Low risk	High risk	Low risk	High risk

Cardiac prognostic risk (GRACE Score⌷)	(n = 1079)	(n = 862)	(n = 995)	(n = 709)
Hospitalization□				
Total hospitalization days#	1.45 (1.36–1.56)	1.13 (1.07–1.19)	1.43 (1.31–1.56)	1.15 (1.07–1.23)
Cardiac-related days#	1.41 (1.29–1.53)	0.97 (0.90–1.04)	1.32 (1.19–1.47)	0.97 (0.87–1.08)
Non-cardiac days	1.54 (1.37–1.74)	1.36 (1.27–1.47)	1.68 (1.44–1.95)	1.31 (1.19–1.43)
				
Ambulatory				
Cardiologist visits	1.16 (1.11–1.21)	1.13 (1.08–1.19)	1.17 (1.11–1.23)	1.11 (1.06 (1.18)
Internist visits	1.12 (1.07–1.17)	1.04 (1.00–1.08)	1.04 (0.99–1.09)	1.14 (1.09–1.20)
GP visits	1.15 (1.12–1.18)	0.97 (0.95–1.00)	1.17 (1.14–1.21)	0.92 (0.90–0.95)
Total ER visits	1.21 (1.07–1.37)	0.98 (0.86–1.11)	1.24 (1.08–1.43)	1.01 (0.87–1.17)
				

	High capacity	Low capacity	High capacity	Low capacity

Functional capacity (DASI score§	(n = 922)	(n = 1019)	(n = 856)	(n = 848)
Hospitalization§				
Total hospitalization days#	1.96 (1.79–2.16)	1.09 (1.04–1.14)	1.56 (1.38–1.77)	1.24 (1.17–1.32)
Cardiac-related days#	1.13 (1.07–1.20)	0.91 (0.78–1.07)	1.17 (0.98–1.40)	1.19 (1.09–1.30)
Non-cardiac days	3.99 (3.51–4.53)	1.04 (0.97–1.12)	2.14 (1.79–2.54)	1.33 (1.22–1.45)
				
Ambulatory				
Cardiologist visits	1.23 (1.15–1.32)	1.14 (1.10–1.18)	1.26 (1.17–1.35)	1.16 (1.11–1.21)
Internist visits	1.33 (1.24–1.43)	1.02 (0.98–1.05)	1.37 (1.28–1.48)	1.09 (1.05–1.13)
GP visits	1.28 (1.23–1.33)	0.98 (0.96–1.00)	1.25 (1.21–1.30)	1.00 (0.97–1.02)
Total ER visits	1.49 (1.24–1.79)	1.00 (0.91–1.11)	1.66 (1.37–2.01)	1.02 (0.91–1.16)

* All relative risks were adjusted for age, sex, income, pre-existing cardiovascular disease, pre-existing non-cardiovascular conditions, in-hospital processes of care, prognostic risk (GRACE and DASI). Abbreviation: AMI: Acute myocardial infarction. Interaction terms were included in models with the complete sample. Depression/GRACE score interactions were statistically significant for the following outcomes when death and recurrent AMI were included (General Practitioner (GP) Visits (P < 0.001) and when death and recurrent AMI were excluded: Total hospitalization days (P < 0.001); Total Cardiac-related hospitalization days (P < 0.001); Non-cardiac hospitalization days (P < 0.001); Cardiologist Visits (P = 0.02); Internist Visits (P = 0.05); GP visits (P < 0.001); and Total ER Counts (P = 0.002). Depression/DASI interactions were significant for the following outcomes when death and recurrent AMI were included: Total hospitalization days (P < 0.001); Non-cardiac hospitalization days (P < 0.001); GP Visits (P < 0.001); and Total Emergency Room (ER) Visits (P < 0.001). The depression/DASI interaction was also significant when death and recurrent AMI were excluded for the following outcomes: Total hospitalization days (P = 0.02); Non-cardiac hospitalization days (P = 0.004); GP Visits (P < 0.001) and Total ER Visits (P = 0.03). Depression status was based on the 9-item depression rating scale.

⌷ Abbreviation: GRACE – Global Registry of Acute Coronary Events.

§ Abbreviation: DASI – Duke Activity Status Index

□ Hospitalization days are a count of total days in hospital over the 18 month follow-up period and can accumulate from multiple hospitalizations.

# Total and cardiac hospitalization results excluded recurrent AMI hospitalizations.

### Mortality and recurrent AMI

There was no significant difference in two-year mortality (HR = 0.92; 95% CI 0.59–1.42), re-AMI (HR = 1.22; 95% CI 0.89–1.66), or in the composite risk of death or recurrent AMI (Adjusted HR = 1.12; 95% CI 0.80–1.57) between patients with and without depressive symptoms.

### Sensitivity analyses

**Figure 2:**
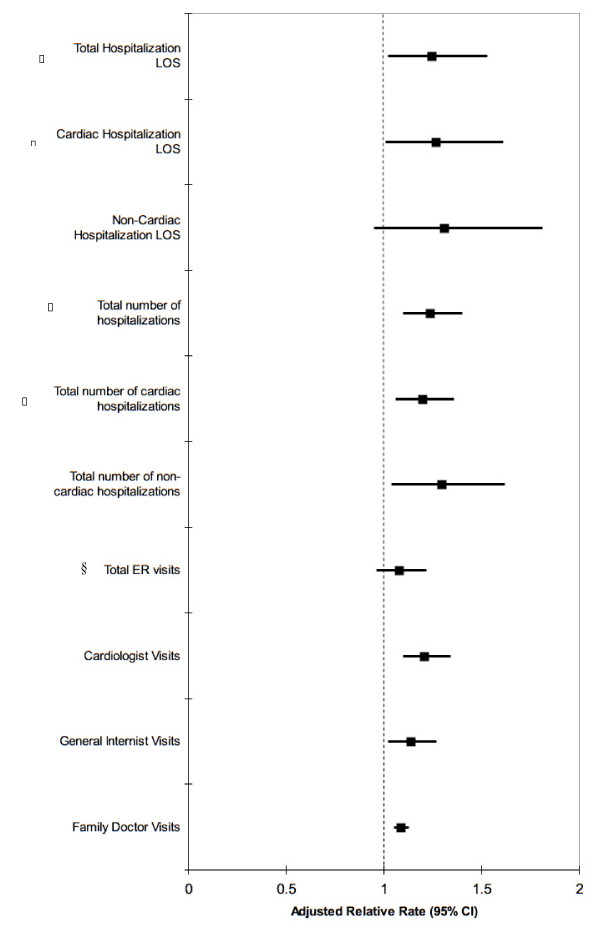
The relative rate of health service consumption attributable to depression* – multiple imputation results. All outcomes were adjusted for age, sex, income, cardiac risk factors, coronary artery bypass graft (CABG), percutaneous, transluminal coronary angiography (PTCA), drugs at discharge, GRACE prognostic index score, and DASI score. Hospitalization days are a count of total days in hospital over the 18-month follow-up period and can accumulate from multiple hospitalizations. * The depression measure is a depression scale containing 9 items from the Brief Carroll Depression Scale (BCDS)(cut-off score of 5). ⌷ Total and cardiac hospitalization results excluded recurrent AMI hospitalizations. §Abbreviation: ER – Emergency Room.

First, the relationship between depression and health service consumption when using the 3 replacement items yielded similar outcomes as those generated with the 9-item BCDRS (see Additional file 1). Second variations in the cutoff scores used to distinguish depressive from non-depressive patients did not alter our results. For example, scores of 4 or 6 using the 9-item BCDRS generated similar results as a score of 5. Similarly, the use of the GUSTO quality of life sub-study depression measure scale yielded similar results as did our 9-item BCDRS. Finally, we used multiple imputation to repeat the analyses to include 888 subjects with missing depression scores, which did not substantively change the results from the complete data analyses (Figure 2).

## Discussion

Depressive symptoms at one-month post-AMI were significantly associated with health service consumption in the 18 months post-AMI. The relationship between health service consumption and depressive symptoms persisted after adjusting for comorbidity and cardiac illness severity. Indeed, the relationship between cardiac health service consumption and depressive symptoms was even greater among those patients with lower as compared with higher cardiac illness severity. In short, the increased likelihood of health service consumption among AMI patients were over and above that expected based on cardiac illness severity alone.

In our sample, depressive symptoms were not associated with increased mortality or recurrent AMI, which is contrary to previous evidence [26,27]. However, the association between depression and cardiovascular outcomes remains controversial [28,29] and the results of this study are not sufficient to address the controversial prognostic importance of depression definitively, especially given inter-study differences in measurement and timing of depression. It is conceivable that measuring depression at one-month post-AMI conferred a survival bias for which multiple imputation methods were unable to fully address. A recent publication measuring depression directly prior to one-month post-AMI found no association between depression and mortality at either time point after adjustment for covariates [30].

Our study demonstrated a significant interaction between depression, prognostic severity and health service consumption, such that higher health service use occurred among patients of lower rather than higher cardiac severity – a distinct contrast to those patients without depressive symptoms whose health service experiences better mirrored their cardiac illness severity levels. Available evidence has demonstrated a treatment-risk paradox whereby service provision is paradoxically most intensive among patients with lowest need [31,32]. Our study suggests that the presence of depressive symptoms might partially mediate the treatment-risk paradox.

Some have hypothesized that mental illness and/or psychological phenomena such as somatization promote health seeking behaviors [6,33]. Our results support such hypotheses. Indeed, in our study, depression-associated increases in the risk of recurrent hospitalization were no longer significant after adjusting for ER visits, suggesting that depression-readmission rates may be attributable to patient health-seeking behaviors more so than physicians' propensity to admit for more discretionary indications[34,35].

Our study has several important clinical and policy implications. Patients with depressive symptoms post-AMI pose a serious challenge to physicians and health care systems. On one hand, such patients are sicker and may be more likely to die following AMI, underscoring the need for closer attentiveness and management[5]. On the other hand, depressive patients consume significant amounts of cardiac health services and may do so disproportionately in relation to objective measures of prognostic or symptomatic burden. Integrated chronic cardiac care programs are well-established and effective interventions for patients post-AMI[36]. Community-based depression interventions have also been shown to be effective at treating depression and improving quality of life[37]. Given the benefits of both chronic vascular disease management[36], depression case-management[37] and the high prevalence of depression in patients post-AMI[38], our study suggests that integrating depression screening and case-management into existing cardiac secondary prevention programs may be effective in improving the quality of life of depressed post-MI patients, and in reducing the apparent mismatch between need and service consumption. The importance of systematic depression screening is further underscored by the low detection rates of depression after AMI[39].

Our study has several noteworthy limitations. First, our scale was designed to ascertain depressive symptoms rather than depressive disorders and was missing 3 items from the original, validated depression rating scale. Our health service consumption findings were similar whether the 9 items or 3 replacement items were used to define depression. The comparability of results with these two depression measures suggests that the two scales are measuring the same construct. Second, depressive symptoms were measured cross-sectionally and at one-month post-AMI. As such, we could not determine the persistence of depressive symptoms over the follow-up interval. The delay in depression post-AMI also imposes a survival bias on the sample. However, survival bias is less of an issue with the main outcome of health service consumption, since service consumption differences are only relevant in those patients who survive long enough to use services. Finally, information regarding depression was missing in just over 30% of the potentially eligible respondent population. However, our sensitivity analyses utilizing model-based multiple imputation to include those patients with missing depression values provide evidence that the missing values did not materially affect our findings.

## Conclusion

In conclusion, our study demonstrated the increased consumption of health services among post-AMI patients with depressive symptoms is independent of comorbidity and cardiac illness severity. Moreover, the interaction between depression, health service utilization, and prognosis such that increased cardiovascular health service consumption patterns among AMI patients with depressive symptoms was most pronounced among those of lower rather than higher cardiac illness severity suggests that cardiac health seeking behaviors among depressive patients may be mediated by psychosocial factors in addition to objective measures of need. Future research must evaluate whether systematic depression detection and integrated chronic disease management systems improve health service efficiency and allow for better alignment between illness severity and health service consumption among such patient populations.

## Competing interests

Dr. Alter serves as a Scientific Director for InterventCanada, a life-style management program. There are no other conflicts of interest to disclose in relation to this study.

## Authors' contributions

PK, PG, WG and DA all conceived of and participated in the design of the study. PK and AC participated in data analysis. PK drafted the manuscript and all other authors contributed to revisions and read and approved the final manuscript.

## Pre-publication history

The pre-publication history for this paper can be accessed here:



## Supplementary Material

Additional File 1 Multivariate* health service consumption rates for the three missing BCDRS items. *Results from multivariate Poisson regression models adjusted for age, sex, income, cardiac risk factors (diabetes, hypertension, hypercholesterolemia, smoking history), medical comorbidities, CABG, PTCA, drugs at discharge (ACE inhibitors, Beta blockers, statins, and nitrates), GRACE score and DASI score and are reported as point estimate with 95% confidence intervals. ⌷ Hospitalization days are a count of total days in hospital over the 18 month follow-up period and can accumulate from multiple hospitalizations. § Total and cardiac hospitalization results excluded recurrent AMI hospitalizations. Click here for file
