# Supplementary material for: The relationship between depressive symptoms, health service consumption, and prognosis after acute myocardial infarction: a prospective cohort study
Source: BMC Health Serv Res. 2008 Sep 30;8:200. doi: 10.1186/1472-6963-8-200 (PMC2576230; doi:10.1186/1472-6963-8-200)
Supplement: Additional File 1 — Multivariate* health service consumption rates for the three missing BCDRS items. *Results from multivariate Poisson regression models adjusted for age, sex, income, cardiac risk factors (diabetes, hypertension, hypercholesterolemia, smoking history), medical comorbidities, CABG, PTCA, drugs at discharge (ACE inhibitors, Beta blockers, statins, and nitrates), GRACE score and DASI score and are reported as point estimate with 95% confidence intervals. ⌷ Hospitalization days are a count of total days in hospital over the 18 month follow-up period and can accumulate from multiple hospitalizations. § Total and cardiac hospitalization results excluded recurrent AMI hospitalizations. [file 1472-6963-8-200-S1.doc]

| Additional file 1: Multivariate* health service consumption rates for the three missing BCDRS items | |
| --- | --- |
|
| Total hospitalization▯§ days | 1.25 (1.20-1.30) |
| Cardiac hospitalization▯§ days | 1.07 (1.01-1.13) |
| Non-cardiac hospitalization▯ days | 1.54 (1.45-1.63) |
| Total number of hospitalizations▯ | 1.10 (0.99-1.22) |
| Total number of cardiac hospitalizations§ | 0.99 (0.89-1.13) |
| Total number of non-cardiac hospitalizations§ | 1.29 (1.09-1.53) |
| Total ER count# | 1.02 (0.93-1.10) |
| Cardiologist visits | 1.12 (1.09-1.16) |
| Internist visits | 0.98 (0.95-1.01) |
| GP visits | 1.06 (1.04-1.08) |
